# Clinician Use of a General-Purpose Large Language Model in Hospital Medicine: A Mixed-Methods Pilot Study

**DOI:** 10.7759/cureus.112223

**Published:** 2026-07-07

**Authors:** Tarek Souaid, Alexander J Ryu, Donna K Lawson, Lindsay Norgaard, Jennifer M Hovell, M. Caroline Burton, Shant Ayanian

**Affiliations:** 1 Hospital Internal Medicine, Mayo Clinic, Rochester, USA

**Keywords:** artificial intelligence, chatgpt, clinical workflow, enterprise ai, hospital medicine, large language model, pilot study

## Abstract

Background

Large language models (LLMs) are entering clinical practice, but real-world use in hospital medicine is not well described.

Methods

We conducted a two-month pilot of a general-purpose LLM application in a hospital medicine division. Two cross-sectional anonymous surveys were administered - one at pilot initiation and one at pilot conclusion.

Results

Response rates were 71.4% (initial) and 48.6% (end of pilot). Reported use was high at both timepoints (18/20 [90.0%] vs 15/17 [88.2%]); median prompts per workday were 2.0 (IQR, 1.0-6.3) and 1.5 (IQR, 1.0-2.0), respectively. Net Promoter Score was +20.0 at initiation and +11.8 at pilot conclusion. Early use emphasized clinical reasoning and administrative tasks; later use shifted toward documentation, summarization, and research work. Efficiency and time savings were the dominant perceived benefit, whereas hallucinations, source transparency, and privacy concerns were the main risks.

Conclusions

General-purpose LLM applications may offer near-term value as supervised tools for lower-risk workflows, but clinical implementation should include structured governance, training, and ongoing evaluation.

## Introduction

Frontier large language models (LLMs) are moving rapidly from experimentation to operational deployment in healthcare. Early commentaries and benchmark studies have highlighted both their promise for communication, summarization, and knowledge work and the need to evaluate real-world clinical use rather than relying on exam-style performance alone [1,2]. Survey data show that, despite the absence of definitive clinical guidelines governing LLM use in practice, healthcare professionals worldwide have already used ChatGPT for a broad range of activities, including generating patient education materials and seeking support for diagnostic and treatment decision-making [3]. Additionally, studies suggest clinicians are generally more comfortable with LLMs in assistive roles than in autonomous decision-making roles [4,5]. At the organizational level, adoption is accelerating: in a recent national survey, 31.5% of US nonfederal acute care hospitals reported current use of generative AI integrated with the electronic health record (EHR) and another 24.7% planned adoption within one year [6]. Still, empirical data describing how hospital-based clinicians actually use enterprise LLMs during early rollout remain limited. We therefore evaluated a two-month pilot of ChatGPT Enterprise (OpenAI, San Francisco, CA, USA) within a hospital internal medicine division using anonymous surveys.

## Materials and methods

We conducted a mixed-methods evaluation of the use of a general-purpose LLM application in a hospital medicine division over two months (January-February 2026). IRB exemption was granted by the Mayo Clinic Institutional Review Board (exemption #25-011731). All clinicians (physicians and advanced practice providers (APPs)) in our hospital’s division of hospital medicine were offered opt-in access to ChatGPT Enterprise (GPT 5.2 family) at study initiation. The study launched with 28 participants; seven additional clinicians joined before the second month, yielding 35 clinicians with at least one month of access by pilot end. Surveys were distributed electronically to all participants via institutional email at each timepoint. An initial survey was distributed after at least one week of access, and an end-of-pilot survey was distributed at pilot completion. Survey completion was voluntary, and all responses were collected anonymously and therefore remained unpaired.

Survey items (Appendix 1) assessed clinical role, self-reported average prompt volume per clinical workday, open-ended use cases, perceived benefits, concerns, and likelihood of recommending the tool for clinical work on a 0-to-10 scale. Net promoter score (NPS) was calculated as the percentage of promoters (scores 9-10) minus the percentage of detractors (scores 0-6), as originally described by Reichheld [7]:

$$\mathrm{NPS} = \%_{\mathrm{Promoters}} - \%_{\mathrm{Detractors}}$$.

We summarized categorical variables as counts and percentages and prompt frequency as median with interquartile range (IQR). Given the anonymous, unpaired surveys design and small sample, we emphasized descriptive analyses. Free-text responses were analyzed using rapid deductive content analysis with iterative consolidation of recurring themes in three domains: use cases, benefits, and concerns. Within each domain, recurring concepts were identified and consolidated into themes. All statistical analyses were performed in R (version 4.5.2; R Foundation for Statistical Computing, Vienna, Austria). Reporting was informed by the STROBE statement for cross-sectional studies and by the Standards for Reporting Qualitative Research principles for the qualitative components [8,9].

## Results

Twenty of 28 eligible clinicians completed the initial survey (71.4%), and 17 of 35 completed the end-of-pilot survey (48.6%). Initial respondents included 13 physicians and seven APPs; end-of-pilot respondents included nine physicians and eight APPs (Table 1).

**Table 1:** Respondent characteristics and survey quantitative outcomes IQR: interquartile range; NPS: net promoter score

Measure	Early survey	End-of-pilot survey
Eligible participants, No.	28	35
Survey respondents, No.	20	17
Response rate, %	71.4	48.6
Physicians, N (%)	13 (65.0)	9 (52.9)
Advanced practice providers, N (%)	7 (35.0)	8 (47.1)
Any enterprise use (>1 prompts/workday), N (%)	18 (90.0)	15 (88.2)
Prompts per clinical workday, median (IQR)	2.0 (1.0-6.2)	1.5 (1.0-2.0)
Recommendation score /10, median (IQR)	7.5 (6.8-9.0)	8.0 (6.0-10.0)
Promoters, N (%)	9 (45.0)	7 (41.2)
Passives, N (%)	6 (30.0)	5 (29.4)
Detractors, N (%)	5 (25.0)	5 (29.4)
Net promoter score	+20.0	+11.8

Reported use remained high throughout the study. The initial 18 of 20 respondents (90.0%) reported at least 1 prompt per clinical workday; at pilot end, 15 of 17 (88.2%) did so. Median prompt volume decreased from a reported average of 2.0 prompts per clinical workday (IQR, 1.0-6.3) initially to 1.5 (IQR, 1.0-2.0) at pilot end. Net Promoter Score remained favorable overall, with NPS of +20.0 at kickoff and +11.8 at pilot end.

**Figure 1:**
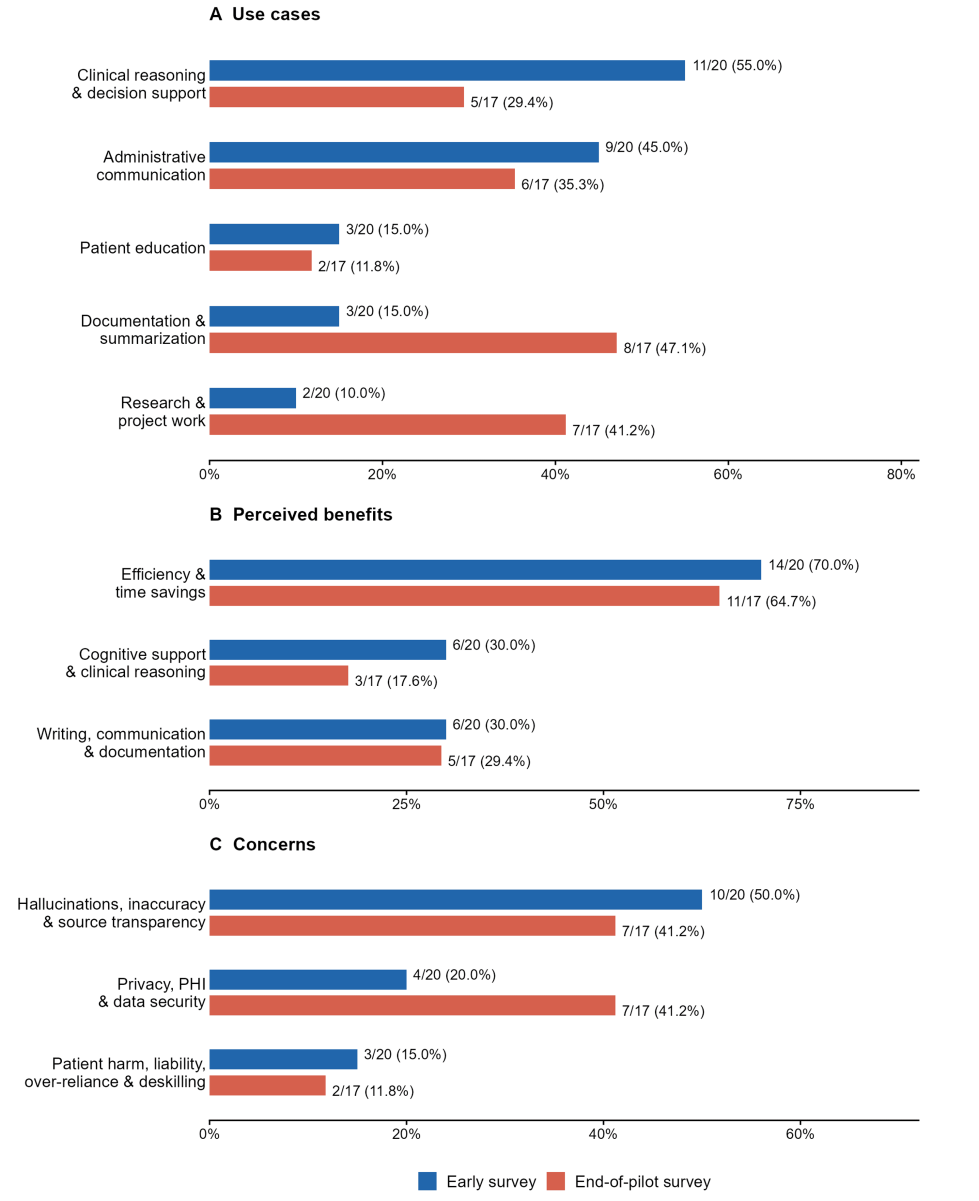
Reported use-case, benefit, and concern themes across pilot timepoints Percentages use survey respondents as denominators (early survey n=20; end-of-pilot survey n=17). Themes were multi-label coded; percentages within a domain can sum to >100%.

The qualitative pattern of use evolved during the pilot (Figure 1). Early use most often involved clinical reasoning and decision support (11/20, 55.0%), followed by administrative communication (9/20, 45.0%). By pilot end, the most frequently cited uses were documentation and summarization (8/17, 47.1%), research or project work (7/17, 41.2%), and administrative communication (6/17, 35.3%). Clinical reasoning was cited less frequently at pilot end than at the start (5/17 [29.4%] vs 11/20 [55.0%]). Patient education was infrequently cited at either timepoint (3/20 [15.0%] at the start and 2/17 [11.8%] at pilot end).

Efficiency and time savings were the most common perceived benefits at both timepoints (14/20 [70.0%] at the start and 11/17 [64.7%] at pilot end). Respondents also described improved writing, communication, and documentation (6/20 [30.0%] and 5/17 [29.4%], respectively) and cognitive support for clinical reasoning (6/20 [30.0%] and 3/17 [17.6%], respectively). Several end-of-pilot respondents explicitly described the enterprise LLM as also useful for non-clinical tasks, including drafting emails, preparing presentations, or organizing project work.

The most common concerns were hallucinations, inaccuracies, and weak source transparency (10/20 [50.0%] at the start and 7/17 [41.2%] at pilot end). Privacy, protected health information, and data security concerns were similarly prominent (4/20 [20.0%] at the start vs 7/17 [41.2%] at pilot end). A smaller number of respondents raised concerns about patient harm, liability, over-reliance, or deskilling (3/20 [15.0%] at the start and 2/17 [11.8%] at pilot end). Several respondents also indicated that for higher-stakes clinical questions they still preferred evidence-linked tools, such as Open Evidence.

Illustrative comments reflected both practical utility and caution. Respondents noted that the tool helped them “create better materials/outlines for patients and their families,” offered “a structured review of clinical reasoning, helping identify gaps in logic,” and enabled more “meaningful feedback” on clinical communication tasks. However, as noted, concerns about “hallucinations,” patient privacy, and the need to “double and triple check the work to ensure accuracy” remained prominent.

## Discussion

In this two-month hospital medicine study, clinicians reported sustained enterprise LLM use, favorable overall recommendation for clinical and administrative tasks, and a consistent perception that the tool improved efficiency. Over time, reported use appeared to shift away from heavier emphasis on direct clinical reasoning and toward documentation, summarization, communication, and research project-related work. This pattern is notable because it suggests practical calibration during early adoption: clinicians may initially explore a broad range of use cases, then concentrate use in tasks where the tool is helpful, fast, and lower risk.

These findings align with prior studies showing that clinicians view LLMs most favorably in assistive roles and value them for synthesis and efficiency rather than for unsupervised decision-making [4,5]. They are also consistent with real-world deployment studies of EHR-integrated generative AI tools, which have found adoption and perceived utility in communication workflows but mixed effects on measured time savings, underscoring the need for local evaluation rather than assumption of benefit [6]. Our findings extend that literature to hospital internal medicine by showing that even without full EHR integration, clinicians identified practical utility in an enterprise-grade HIPAA-compliant LLM.

The risk signal in this study is also important to highlight. Concerns about hallucinations, source transparency, and privacy remained notable, and privacy concerns increased by pilot end, despite participants having been informed of the HIPAA-compliant nature of the enterprise account. These concerns are aligned with broader calls for careful testing, governance, and post-deployment monitoring as health systems adopt generative AI [1,2,6,10,11]. For hospital medicine programs considering enterprise LLM access, our data support positioning these tools as supervised adjuncts for lower-risk language tasks rather than as stand-alone clinical decision aids. Prior to broader implementation, staff training should explicitly address prompt hygiene, verification against evidence-linked resources, and the difference between acceptable assistance and unsafe reliance.

Our reported perception of efficiency as the dominant benefit is consistent with findings from prior general-purpose LLM reports in healthcare settings, which similarly describe strong clinician interests in time-saving and synthesis applications [12,13]. The shift in use emphasis we observed (from clinical reasoning early in the pilot toward documentation, summarization, and administrative tasks by pilot end) is also aligned with findings from other short-term clinical AI evaluations describing concerns for fake clinical evidence [14]. Concerns about hallucinations and inaccuracy, as mentioned above, mirror rates documented across multiple clinical settings and model types, and underscore that this challenge is not specific to enterprise implementations [13,15]. The persistence of privacy concerns despite the use of a HIPAA-compliant account has similarly been noted in other healthcare AI studies and likely reflects a broader unease that persists independent of specific technical safeguards [12,14]. Collectively, these convergent patterns suggest that the adoption dynamics and risk perceptions we identified are likely representative of early-adoption experiences in hospital medicine and comparable acute-care settings.

This study has limitations. It was conducted in a single division with a small sample and modest response rates, particularly at pilot end. Surveys were anonymous and unpaired, preventing within-person comparisons. The short-term follow-up period may have limited the ability to assess the durability of observed outcomes in the long term. Because clinician participation was voluntary, selection bias may have been introduced, as participants may differ systematically from nonparticipants in their experiences, perspectives, or engagement with LLMs. Results relied on self-report rather than objective audit logs, time-motion data, or clinical outcomes. In addition, theme coding was based on short free-text survey responses rather than in-depth interviews.

## Conclusions

In summary, this pilot study suggests that enterprise LLMs may have immediate value for hospitalists and advanced practice providers in drafting, summarization, communication, and other lower-risk clinical workflow tasks. Going forward, our division intends to critically assess the value of broadening access to similar tools. However, favorable early sentiment does not equate evidence of safety or effectiveness in higher-stakes clinical reasoning. Health systems should pair access to such tools with governance, training, and ongoing evaluation, always grounded in real-world use.

## Appendices

Appendix 1

**Table 2:** Pilot Survey Instrument

Item	Survey question	Response format
1	What is your role in Hospital Internal Medicine?	Multiple choice: Physician; NPPA
2	What did you use ChatGPT Enterprise for on your past clinical week?	Free-text response
3	On average, for this past clinical week, how many prompts per clinical workday did you send to ChatGPT Enterprise?	Numeric free-text response
4	What benefits did you perceive using ChatGPT Enterprise? Please quantify when possible (e.g., time saved on specific tasks). Enter “0” if no benefit was perceived.	Free-text response
5	What risks or concerns do you have about using ChatGPT Enterprise in your practice?	Free-text response
6	How likely are you to recommend ChatGPT Enterprise to your colleagues for clinical work?	0-10 Likert scale, where 0 = not at all likely and 10 = extremely likely
